# Circulating Growth Factors and Cytokines Correlate with Temperament and Character Dimensions in Adolescents with Mood Disorders

**DOI:** 10.3390/brainsci15020121

**Published:** 2025-01-26

**Authors:** Maria Terczynska, Weronika Bargiel, Maksymilian Grabarczyk, Tomasz Kozlowski, Przemyslaw Zakowicz, Dawid Bojarski, Karolina Wasicka-Przewozna, Pawel Kapelski, Aleksandra Rajewska-Rager, Maria Skibinska

**Affiliations:** 1 The Student Scientific Society of Poznan University of Medical Sciences, Student’s Research Group “Biological Psychiatry”, Department of Psychiatric Genetics, Poznan University of Medical Sciences, 60-806 Poznan, Poland; 2 Collegium Medicum, University of Zielona Gora, 65-417 Zielona Gora, Poland; przemek@zakowicz.eu; 3 Center for Children and Adolescent Treatment in Zabor, 66-003 Zabor, Poland; 4 Independent Researcher, 60-806 Poznan, Poland; 5 Department of Psychiatric Genetics, Poznan University of Medical Sciences, 61-701 Poznan, Polandrajrager@ump.edu.pl (A.R.-R.)

**Keywords:** adolescent mood disorders, major depressive disorder, bipolar disorder, Temperament and Character Inventory, peripheral biomarkers

## Abstract

**Background/Objectives**: The incidence of mood disorders in adolescents is increasing. Bipolar disorder is often misdiagnosed in the early stages of the disease due to the prevalence of depressive symptoms, while manic episodes occur later. Identifying predictors of diagnosis conversion could facilitate timely and appropriate treatment. Our study aimed to find correlations of selected peripheral protein levels with temperament and character traits in adolescents diagnosed with major depressive disorder and bipolar disorder. **Methods**: A group of adolescents and young adults diagnosed with major depressive disorder (MDD, *n* = 50) or bipolar disorder (BD, *n* = 24) was enrolled in the study during the exacerbation of symptoms and followed up over two years. Diagnosis conversion from MDD to BD was monitored. The Temperament and Character Inventory was applied, and BDNF, proBDNF, EGF, MIF, SCF, S100B, TNF-alpha, and IL-8 serum levels were measured. Spearman’s rank correlation analysis was conducted. **Results**: We found different patterns of correlations in MDD (TNF-alpha, IL-8, EGF, S100B with reward-dependence, self-directedness, and empathy) and BD (BDNF and EGF with persistence novelty-seeking and self-transcendence). Significant correlations were found in a group with diagnosis conversion. **Conclusions**: The findings of our study have the potential to significantly impact our understanding and treatment of mood disorders. Correlations obtained in the subgroup with diagnosis conversion may contribute to the development of prognostic markers in the future. Evaluating temperament and character traits alongside established biomarkers may offer a valuable method for predicting the conversion of mood disorders in adolescents, facilitating early and effective pharmacotherapy.

## 1. Introduction

The incidence of mood disorders worldwide is approximately 20% [1]. In almost half of young people with a primary diagnosis of major depressive disorder (MDD), it converts to bipolar disorder (BD) [2]. Markers of the diagnosis conversion from MDD to BD would enable faster application of appropriate treatment and, thus, better prognosis for the patient [3]. Unfortunately, clinical predictors or biomarkers of diagnosis conversion have not been established. Combining these two approaches would benefit with better sensitivity and specificity.

The Temperament and Character Inventory (TCI) is a questionnaire that provides a comprehensive biopsychosocial model of personality. According to Cloninger, personality consists of temperament and character. Temperament is defined by automatic responses to perceived stimuli and personal differences in associative learning. It is divided into the following four dimensions: harm-avoidance (HA), novelty-seeking (NS), reward-dependence (RD), and persistence (P). HA is a tendency to inhibit or avoid action in response to negative stimuli. It is related to an increase in serotonergic neurotransmission and is composed of four sub-dimensions as follows: fear of uncertainty, shyness, fatigability, and anticipatory worry. Novelty-seeking (NS) is related to decreased dopamine activity and involves active responding and seeking new stimuli. NS consists of exploratory excitability, impulsiveness, extravagance, and disorderliness. Reward-dependence (RD) is a propensity to continue a behavior in response to positive reinforcement. RD is associated with noradrenergic transmission and includes sentimentality, attachment, and dependence. Persistence is the tendency to sustain a particular activity despite fatigue and frustration. It has not been linked to any neurotransmitter in Cloninger’s model [4].

Character encompasses values, goals, and self-conscious emotions shaping individual conduct. It is divided into the following three dimensions: self-directedness (SD), cooperativeness (C), and self-transcendence (ST). Self-directedness (SD) is a person’s ability to adapt his behavior to a given situation per his objectives and values. SD includes five features as follows: responsibility, purposefulness, resourcefulness, self-acceptance, and enlightened second nature. Cooperativeness (C) is expressed in approving other people’s behavior and identifying with them. It consists of social acceptance, empathy, helpfulness, compassion, and integrated conscience. Self-transcendence (ST) is a person’s patience, spirituality and ability to forget oneself and identify with more significant ideas. Within it, three sub-dimensions are distinguished as follows: self-forgetfulness, transpersonal identification, and spiritual acceptance. While temperament is highly heritable and strongly influenced by genetic factors, character traits are, to a greater extent, influenced by environmental stimuli [4,5,6].

In addition to the TCI questionnaire, there are several clinical instruments used in the study of temperament and character, used in groups of psychiatric patients, in the spectrum of mental disorders, and healthy individuals. The most commonly used include the Temperament Evaluation of Memphis, Pisa, Paris, and San Diego-Interview (TEMPS-I) and the self-administered questionnaire TEMPS-A [7] and the NEO Personality Inventory (NEO-PI-3) [8]. Concepts of affective temperaments measured with TEMPS-A include anxious, cyclothymic, depressive, and irritable temperaments, which are grouped into emotional instability factor, and hyperthymic affective temperament, which is considered as emotionally stable [9]. Based on the available research questionnaires, attempts are being made to create a comprehensive model of temperament and character [10]. Integrating clinical tools with objectively measured peripheral biomarkers will enhance accurate diagnosis and timely pharmacological treatment.

Among the potential biological markers of mood disorders, neurotrophins, cytokines, and growth factors are often studied. In our research, we used brain-derived neurotrophic factor (BDNF) and its precursor (proBDNF). BDNF, a pivotal regulator of the structural and functional plasticity of neurons in the central nervous system (CNS), is essential for their growth and differentiation. It also influences immune–inflammatory pathways in the CNS [11,12]. In contrast, proBDNF, the precursor of BDNF, exhibits opposing properties. ProBDNF causes neuronal apoptosis [13], and inhibits synaptic transmission and plasticity [14]. Another protein with trophic properties is S100 calcium-binding protein B (S100B), which is produced mainly by glial cells. S100B shows trophic and proliferative effects on neurons and astrocytes when acting intracellularly and at nanomolar concentrations. In contrast, when secreted extracellularly and at micromolar concentrations, it exhibits proapoptotic effects on CNS cells [15,16]. The potential of neurotrophins as biological markers is a significant area of study, with implications for our understanding of mood disorders.

Our investigation into growth factors led us to focus on the epidermal growth factor (EGF) and stem cell factor (SCF). EGF plays a significant role in the proliferation, development, and maturation of neurons and glial cells during neurogenesis and throughout the adult brain [17,18]. Similarly, SCF’s stimulation of neuronal growth, proliferation, and protection, as well as its responsibility for the migration of neural progenitor cells and neuron–glia interactions, is an engaging aspect of our research [19].

Among the cytokines, we chose Tumor Necrosis Factor-α (TNF-α), Interleukin-8 (IL-8), and Migration Inhibitory Factor (MIF). Macrophages mainly produce TNF-α, also secreted by glial cells and neurons. In the CNS, it plays a role in inflammatory processes, typically acting as a pro-inflammatory mediator. TNF-α affects neuronal function, the amount of available neurotransmitters, and myelin synthesis, increasing blood–brain barrier permeability [20]. IL-8 is released in the brain by macrophages, brain microglia, and astrocytes. Depending on its concentration, it may have pro- or anti-inflammatory properties [21]. Studies have shown that Il-8 is associated with the pathophysiology of mood disorders [22]. MIF has biological features of cytokines and hormones [23]. It is secreted by the pituitary and adrenal glands following activation of the hypothalamic–pituitary–adrenal (HPA) axis and is linked to neurogenesis [24].

This study aimed to examine correlations between the serum levels of neurotrophic factors, growth factors, and cytokines: brain-derived neurotrophic factor (BDNF) and its precursor (proBDNF), epidermal growth factor (EGF), stem cell factor (SCF), migration inhibitory factor (MIF), S100 calcium-binding protein B (S100B), Tumor Necrosis Factor-alpha (TNF-alpha), and Interleukin 8 (IL-8) with Temperament and Character Inventory dimensions in adolescents diagnosed with mood disorders—major depressive disorder (MDD) or bipolar disorder (BD). TCI and protein level measurements were performed during the exacerbation of the disease, in the depression or hypomanic/manic episode (baseline visit). Monitoring of diagnosis conversion from MDD to BD during two years of follow-up was performed. The study aimed to decipher correlations of temperament and character dimensions with selected circulating proteins with regard to the diagnosis of major depressive disorder or bipolar disorder, with particular emphasis on potential predictors of diagnosis conversion. We analyzed the total patients’ group and divided them into MDD and BD subgroups. A study in the patients’ group with diagnosis conversion was also performed.

## 2. Materials and Methods

### 2.1. Participants

A comprehensive description of the study group has been published previously [25]. Briefly, we conducted a two-year follow-up study involving *n* = 74 adolescents and young adults diagnosed with mood disorders. Their diagnoses of major depressive disorder (MDD) or bipolar disorder (BD) were confirmed with ICD-10 and DSM-IV criteria. The clinical evaluations of the mood state were made at the baseline and during follow-up visits which occurred at 3 months, 6 months, 1 year, and 2 years. Adolescent patients were interviewed using the Kiddie Schedule for Affective Disorders—Present and Lifetime (KSADS-PL) [26], while adult patients were assessed with a structured clinical interview for DSM-IV (SCID) [27]. These assessments were used to evaluate lifetime psychiatric disorders and to monitor current mood episodes. The Hamilton Depression Rating Scale (HDRS-17) [28] and Young Mania Rating Scale (YMRS) were utilized to assess the severity of depressive and manic symptoms [29]. Participants received treatment at the Department of Child and Adolescent Psychiatry and Adult Psychiatry Department of Poznan University of Medical Sciences. Written informed consent was obtained from all participants and/or their legal guardians. The study protocol was approved by The Poznan University of Medical Sciences Ethics Committee (no. 362/11). During the study, all patients received pharmacological treatment based on their mood episodes. MDD patients were monitored for the occurrence of hypomanic/manic episodes throughout the follow-up period.

### 2.2. Temperament and Character Measurement

The Temperament and Character Inventory (240-item version), developed by Cloninger, was administered to the patients. The temperament dimensions of the TCI include novelty-seeking (NS), harm-avoidance (HA), reward-dependence (RD), and persistence (P). The three character dimensions are self-directedness (SD), cooperativeness (C), and self-transcendence (ST). Each dimension (except of persistence) consist of three to five subcomponents [6]. Missing responses (2%) in the TCI questionnaire were replaced with a value of 0.5.

### 2.3. Enzyme-Linked Immunosorbent Assays of Serum BDNF, proBDNF, EGF, MIF, SCF, S100B, IL-8, and TNF-Alpha Levels

Venous blood samples were collected in anticoagulant-free tubes in the morning after an overnight fast. Following a 1-h incubation at room temperature, serum was separated by centrifugation, aliquoted, and stored at −70 °C until analysis. Enzyme-linked immunosorbent assays (ELISA) were conducted using BDNF, proBDNF, EGF, MIF, and SCF with the DuoSet ELISA Development Kits (R&D Systems, Minneapolis, MN, USA). This was performed according to the manufacturer’s instructions, with minor modifications as previously described [30,31]. A human high-sensitivity magnetic luminex performance assay (R&D System, Minneapolis, MN, USA) was utilized to determine the levels of IL-8 and TNF-alpha, as detailed elsewhere [32]. All samples and standards were analyzed in duplicates, with intra-assay variability and inter-assay variability being less than 5% and 10% CV, respectively, for each protein studied.

### 2.4. Mature BDNF (mBDNF) and mBDNF/proBDNF Ratio Estimation

We previously demonstrated that the Human BDNF DuoSet ELISA kit detects both mature BDNF and 100% of proBDNF [31]. To calculate the concentration of mature BDNF (mBDNF), we subtracted the levels of proBDNF from the total BDNF (tBDNF) concentrations. This resulted in the concentration of mBDNF, which we used to estimate the mBDNF/proBDNF ratio (rBDNF).

### 2.5. Statistical Analyses

The Kolmogorov–Smirnoff test was conducted to assess the normality of the data. All the studied protein concentrations exhibited a non-normal distribution; therefore, nonparametric Spearman’s correlation was used in the analyses. The significance level was set at *p* < 0.05. The statistical analyses were performed using Statistica v13 software (StatSoft, Krakow, Poland). A power of ≥80 in the correlation analysis was achieved with the study group sizes: *n* = 74 (all patients) with R ≥ 0.32, *n* = 50 (MDD) with R ≥ 0.39, *n* = 24 (BD) with R ≥ 0.55, and *n* = 14 (group with diagnosis conversion) with R ≥ 0.68 (https://sample-size.net/correlation-sample-size/, accessed at 28 December 2024).

## 3. Results

The clinical and demographic characteristics of the studied group were described previously, as well as comparisons of the TCI variables between MDD and BD subgroups at baseline [25]. Analyses of the studied protein levels with clinical variables were presented elsewhere [30,31,32]. During two-year follow-up observation, 14 patients changed diagnosis from major depressive disorder to bipolar disorder. In Table 1, we present the demographic characteristics and protein levels in the studied groups.

### 3.1. Correlations of BDNF, proBDNF, EGF, MIF, SCF, S100B, TNF-Alpha, and IL-8 Serum Levels with Temperament and Character Inventory Dimensions in MDD + BD Patients Group

We found the following correlations of the studied proteins with temperament and character traits: IL-8 and TNF-alpha with self-directedness (SD) (R = 0.36, *p* = 0.003 and R = 0.39, *p* < 0.001, respectively); TNF-alpha with harm-avoidance (HA) (R = −0.32, *p* = 0.01) and reward-dependence (RD) (R = 0.35, *p* = 0.005); IL-8 with anticipatory worry (HA1) (R = −0.38, *p* = 0.002), purposefulness (SD2) (R = 0.37, *p* = 0.002), and enlightened second nature (SD5) (R = 0.008); and TNF-alpha with anticipatory worry (HA1) (R = −0.34, *p* = 0.007), fatigability (HA4) (R = −0.33, *p* = 0.008), attachment (RD2) (R = 0.45, *p* < 0.001), purposefulness (SD2) (R = 0.53, *p* < 0.001), resourcefulness (SD3) (R = 0.38, *p* = 0.002), and enlightened second nature (SD5) (R = 0.38, *p* = 0.002). S100B negatively correlated with empathy (C2) (R = −0.35, *p* = 0.004). Presented correlations achieved power ≥ 80%. We did not find any other significant correlations with power ≥ 80% between tBDNF, mBDNF, proBDNF, rBDNF, EGF, MIF, and SCF and temperament or character dimensions in the MDD + BD group. The results of correlations with power ≥ 80% are presented in Table 2. All correlation results are included in Supplementary Table S1.

### 3.2. Correlations of BDNF, proBDNF, EGF, MIF, SCF, S100B, TNF-Alpha, and IL-8 Serum Levels with Temperament and Character Inventory Dimensions and Its Subcomponents in MDD Patients Group

In the MDD patients’ subgroup, IL-8 and TNF-alpha positively correlated with the reward-dependence (RD) dimension (R = 0.40, *p* = 0.008 and R = 0.50, *p* < 0.001, respectively), and attachment (RD2) (R = 0.44, *p* = 0.003 and R = 0.53, *p* < 0.001, respectively). EGF correlated with self-acceptance (SD4) (R = 0.04, *p* = 0.004). TNF-alpha correlated with resourcefulness (SD3) (R = 0.39, *p* = 0.01), and S100B with empathy (C2) (R = −0.43, *p* = 0.003). We did not find any other significant correlations with power ≥80% between tBDNF, mBDNF, proBDNF, rBDNF, MIF, or SCF and temperament or character dimensions in the MDD group. The results of correlations with power ≥ 80% are presented in Table 2. All correlation results are included in Supplementary Table S2.

### 3.3. Correlations of BDNF, proBDNF, EGF, MIF, SCF, S100B, TNF-Alpha, and IL-8 Serum Levels with Temperament and Character Inventory Dimensions in BD Patients Group

In the BD patients’ group, EGF positively correlated with novelty-seeking (NS) (R = 0.59, *p* = 0.02), rBDNF with self-transcendence (ST) (R = 56, *p* = 0.006), tBDNF (R = 0.58, *p* = 0.003) and mBDNF (R = 0.7, *p* < 0.001) with persistence (PS). rBDNF correlated with disorderliness (NS4) (R = 0.69, *p* < 0.001) and transpersonal identification (ST2) (R = 0.6, *p* = 0.003).

We did not find any other significant correlations with power ≥ 80% between proBDNF, MIF, SCF, S100B, TNF-alpha, or IL-8 and temperament or character dimensions in the BD group.

The results of correlations with power ≥ 80% are presented in Table 2. All correlation results are included in Supplementary Table S3, along with other significant results with power < 80%.

### 3.4. Correlations of BDNF, proBDNF, EGF, MIF, SCF, S100B, TNF-Alpha, and IL-8 Serum Levels with Temperament and Character Inventory Dimensions in the Group with Diagnosis Change from MDD to BD

We obtained significant correlations in the subgroup of patients with diagnosis conversion from MDD to BD, although, due to a small number of patients (*n* = 14), the power of these correlations did not achieve 80%. The following correlations were detected: TNF-alpha with self-directedness (SD) (R = 0.66, *p* = 0.02), attachment (RD2) (R = 0.65, *p* = 0.02), and helpfulness (C3) (R = 0.61, *p* = 0.03); tBDNF (R = 0.6, *p* = 0.02) and mBDNF (R = 0.66, *p* = 0.01) with self-acceptance (SD4); proBDNF and rBDNF with disorderliness (NS4) (R = 0.55, *p* = 0.04 and R = −0.56, *p* = 0.04, respectively), and IL-8 with empathy (C2) (R = −0.59, *p* = 0.04).

We did not find any other significant correlations between EGF, MIF, SCF, or S100B and temperament or character dimensions in the BD group.

The results of correlations of the studied proteins with TCI results in the group with diagnosis change are presented in Supplementary Table S4.

## 4. Discussion

In this study, we detected different patterns of correlations between temperament and character traits with the studied peripheral protein levels. Some correlations in the whole studied group reflect correlations in the MDD group (which is larger), and encompass mainly interleukins. In the BD group we found positive correlations of growth factors BDNF and EGF. We did not detect any correlations of MIF and SCF in our study.

In the whole studied group (MDD + BD), we found a negative correlation of TNF-alpha with the harm-avoidance (HA) dimension and fatigability (HA4), and both TNF-alpha and IL-8 with anticipatory worry (HA1). A positive correlation of both interleukins with character dimension self-directedness (SD), and its sub-dimensions purposefulness (SD2) and enlightened second nature (SD5). Only TNF-alpha correlated with resourcefulness (SD3). S100b correlated negatively with empathy (C2).

In the MDD group, we detected positive correlations between TNF-alpha and IL-8 with the reward-dependence (RD) dimension, and its sub-dimension attachment (RD2). We found the following correlations with sub-dimensions of self-directedness: EGF and self-acceptance (SD4), TNF-alpha with purposefulness (SD2), and resourcefulness (SD3). S100b correlated negatively with emphaty (C2).

In the BD group, we detected positive correlations of EGF with novelty-seeking (NS), while total BDNF and mature BDNF correlated with persistence (PS). BDNF/proBDNF ratio (rBDNF) was associated with self-transcendence (ST), disorderliness (NS4), and transpersonal identification (ST2).

Our previous study showed higher scores in harm-avoidance (HA) dimensions along with anticipatory worry (HA1) and fatigability (HA4) in MDD patients, while BD patients have higher scores in self-directedness (SD), purposefulness (SD2), and enlightened second nature (SD5). Self-forgetfulness (ST1) and transpersonal identification (ST2) were also higher in BD patients [25]. We can observe different patterns of temperament and character dimensions between MDD and BD adolescent patients, as well as characteristic protein correlations with TCI scores (in MDD with reward-dependence, self-directedness, and empathy, in BD with novelty-seeking, self-transcendence, and persistence).

The most recent genome-wide association study (GWAS) revealed 736 gene loci in 51 SNP sets significantly associated with temperament, with TNF-based resilience, neurotrophin, and serotonin–cytokine interaction sets [33]. Until now, there have been limited studies on circulating proteins’ correlations with temperament and character dimensions in mood disorders and healthy populations.

### 4.1. BDNF

The results of BDNF studies in the context of temperament and character dimensions of TCI, while varying regarding the study group and specific association/correlation, hold great potential for future research and applications in the field of psychology and neuroscience.

Genetic studies revealed an association of BDNF rs6265 polymorphism with self-transcendence in BD patients [34]. In healthy females, the association of Val66Met polymorphism with reward-dependence was found [35]. Novelty-seeking was associated with BDNF rs61888800 polymorphism in depression [36]. Kazantseva et al. (2014) found an association of a specific haplotype block within the BDNF gene with higher persistence in a large cohort of young adults and the moderating effect of gender [37].

Only a few studies exist on circulating BDNF levels and temperament and character dimensions. In healthy populations, a negative correlation has been observed between harm-avoidance (HA) and BDNF serum [38] or plasma [39] levels. Plasma BDNF levels were positively correlated with self-directedness (SD) scores in healthy Japanese subjects [39]. Other studies did not reveal the relationship between TCI scores and BDNF levels in healthy subjects [40]. Nomoto et al. (2015) discovered a negative correlation of serum BDNF with self-directedness (SD) in major depressive disorder [41]. Our team found a negative correlation between the BDNF serum level and reward-dependence (RD) in adolescent girls diagnosed with anorexia nervosa [42].

The presented results have significant implications for understanding the influence of BDNF serum levels on persistence (P). We detected correlations of mBDNF/proBDNF ratio with self-transcendence (ST), disorderliness (NS4), and transpersonal identification (ST2).

The BDNF/proBDNF ratio has not been studied as extensively in psychiatric disorders compared to the level of BDNF itself. Our results suggest that the balance between BDNF and proBDNF may offer greater predictive value than measuring BDNF alone.

### 4.2. TNF-Alpha and IL-8

The presented results show that IL-8 and TNF-alpha correlate with reward-dependence in people with depression. TNF-alpha and IL-8 may increase reward-dependence by affecting the anterior cingulate cortex (ACC). fMRI studies showed a relationship between ACC activity and reward-dependence [43]. Cytokines activate the ACC [44] by increasing the concentration of glutamate [45,46]. ACC is related to social pain, processes rejection-related distress, and negative affect [47,48], so a change in its activity may cause increased reward-dependence.

Cloninger’s biopsychosocial model of personality posits that reward-dependence is associated with low levels of norepinephrine (NE) [4]. Studies have shown that TNF-alpha regulates NE metabolism in mice during inflammation [49,50]. In addition, the effect of the antidepressant desipramine is associated with a change in TNF-alpha activity from inhibiting to promoting NE release [51]. Thus, TNF-alpha may influence reward-dependence by affecting the amount of free NE.

We noticed that in the group suffering from depression, there were also positive correlations between IL-8 and SD and also between TNF-alpha and self-directedness (SD). It has been shown that TNF-alpha increases the expression and activity of serotonin transporter (SERT) [52]. It has also been noted that higher scores in the character SD dimension are associated with higher transcriptional activity of the SERT gene and higher serotonin uptake of this protein [53,54,55]. Thus, it can be hypothesized that TNF-alpha increases SD through increased SERT expression and activity. Moreover, the association of cytokines with SD may result from their effects on specific brain regions. Self-directedness scores correlate negatively with metabolism in the right supramarginal gyrus in healthy adults [56], and elevated levels of soluble tumor necrosis factor receptor-1 (sTNF-R1) correlate negatively with gray matter volume in the supramarginal gyrus [57]. The right supramarginal gyrus is the brain region responsible for recognizing emotions and overcoming emotional egocentrism [58,59]. Hence, TNF-alpha may affect the activity of this brain area.

Opposite correlations were obtained in our study of TNF-alpha with TCI dimensions—negative with harm-avoidance (HA), characterized by increased serotonin, and positive with reward-dependence (RD) connected with decreased noradrenaline might reflect processes in the specific brain areas involved in the neurobiology of temperament.

### 4.3. EGF

In the MDD subjects, we also found a correlation between EGF levels and performance on the self-directedness dimension. Dávila et al. showed that the Val158Met polymorphism in the catechol-O-methyltransferase (COMT) gene causes a decrease in self-directedness in people with bipolar disorder (49). It can be concluded that a reduction in COMT activity is associated with a decrease in SD. Moreover, increased EGF mRNA levels resulted in increased COMT levels in several brain regions of the transgenic mice [60]. Thus, there may be some relationship between the effect of EGF on COMT activity and outcomes in SD, but there is a lack of literature to confirm this.

Gil et al. (2003) showed that EGF increases SERT activity [61], consistent with the studies already cited above, showing that higher SERT activity is associated with higher scores in SD [53,54,55].

This study observed a strong positive correlation between EGF concentration and novelty-seeking (NS) in BD patients. This finding suggests a potential influence of EGF on NS, a trait related to dopaminergic transmission. Studies indicate that EGF affects the development of midbrain dopaminergic neurons [62] and dopamine metabolism [60], which may influence NS. This potential influence of EGF on novelty-seeking opens up a broader area of research and implications for the understanding of bipolar disorder.

### 4.4. S100B

A genetic study of two polymorphisms in the S100B gene revealed an association with self-directedness [63]. The S100B protein might be involved not only in pathological brain conditions but also in normal behavior. In our study, S100B was negatively correlated with empathy (C2), a sub-dimension of the cooperativeness character trait. Further studies focused on the role of S100B in temperament and character modulation are needed.

### 4.5. Correlations in the Group with Diagnosis Conversion

This study appears to be the first to examine the correlations between protein levels and temperament and character traits concerning the conversion of diagnoses in mood disorders. Although the patient group was small, our findings indicate that further research with larger cohorts is necessary. Our results suggest that combining peripheral protein levels with psychological assessments may have potential as a prognostic biomarker of diagnosis conversion from MDD to BD in the early phase of bipolar disorder.

### 4.6. Limitations

The study’s main limitations include a low sample size, particularly in the subgroup with diagnosis conversion. None of the significant correlations in this group reached the 80% power threshold. Additionally, confounding factors such as pharmacological treatment, variations in disease severity, or lifestyle factors were not included in the analysis. The absence of a control group does not allow a cross-sectional comparison of the TCI results and their correlations with biological factors between patients and healthy individuals. Furthermore, the study did not include repeated measurements of the analyzed protein, not allowing the observation of temporal changes in potential biomarker levels. Finally, the Temperament and Character Inventory is a self-reported measure, which may affect the accuracy of ratings due to potential social desirability bias.

The results of this research should be interpreted carefully as a preliminary study. Future studies should involve larger cohorts of patients and a control group.

## 5. Conclusions

The presented study results reveal significant correlations between the personality and character traits evaluated using the Temperament and Character Inventory and serum concentrations of selected neurotrophins, growth factors, and cytokines in adolescents with mood disorders. We discovered different patterns of correlations between groups diagnosed with major depressive disorder (MDD) and bipolar disorder (BD). In the MDD group, inflammatory factors were prevalently correlated with reward-dependence and self-directedness, while trophic factors were associated with persistence, novelty-seeking, and self-transcendence in BD subjects. Correlations obtained in the subgroup with diagnosis conversion may contribute in the future to the prognostic markers’ development. The findings of this study, if confirmed in larger populations, could significantly enhance our understanding and treatment of mood disorders. If the initial results of this study are validated with a larger group, integrating the assessment of temperament and character traits with peripheral biomarkers could become a valuable method for predicting the diagnosis conversion of mood disorders among adolescents. Early and accurate diagnosis of diseases is essential for timely treatment.

## Figures and Tables

**Table 1:** Clinical and demographic characteristic of the studied group. Mean serum protein levels (pg/mL).

	MDD + BD	MDD	BD
*n*	74	50	24
Female/Male	54/20	38/12	16/8
Mean age (±SD)	18.49 (±3.34)	18.31 (±3.28)	18.95 (±3.52)
Mean age at illness onset (±SD)	16.82 (±2.68)	16.8 (±2.94)	16.86 (±2.05)
Drug-free yes/no	25/49	21/29	4/20
Inpatient/outpatient	56/18	35/15	21/3
Family history of any psychiatric disorder yes/no	46/28	35/15	11/13
Family history of affective disorder yes/no	37/37	29/31	8/16
HDRS-17	14.88 (±8.17)	19.28 (±5.39)	5.71 (±4.48)
YMRS	6.12 (±8.63)	0.96 (±1.48)	16.88 (±7.29)
tBDNF (pg/mL) (mean ± SD)	25,265.07 (±8083.81)	24,107.78 (±6437.74)	27,676.1 (±10,494.6)
mBDNF (pg/mL) (mean ± SD)	23,020.96 (±8372.06)	21,748.73 (±6602.92)	25,671.43 (±10,896.38)
proBDNF (pg/mL) (mean ± SD)	2244.11 (±2232.92)	2359.04 (±2320.40)	2004.67 (±2065.33)
rBDNF (mean ± SD)	24.06 (±24.94)	24.20 (±26.16)	23.75 (±22.50)
EGF (pg/mL) (mean ± SD)	224.73 (±152.16)	222.92 (±148.87)	228.48 (±162.01)
MIF (pg/mL) (mean ± SD)	1864.62 (±1056.00)	1928.81 (±1148.07)	1730.90 (±838.88)
SCF (pg/mL) (mean ± SD)	157.49 (±103.35)	163.70 (±118.33)	144.78 (±63.08)
S100B (pg/mL) (mean ± SD)	150.41 (±74.76)	149.28 (±64.71)	152.69 (±93.29)
TNF-alpha (pg/mL) (mean ± SD)	7.35 (±2.78)	6.85 (±2.42)	8.30 (±3.23)
IL-8 (pg/mL) (mean ± SD)	12.57 (±14.70)	9.87 (±9.83)	17.85 (±20.54)

HDRS-17—Hamilton Depression Rating Scale (17 item); YMRS—Young Mania Rating Scale; BDNF—Brain-Derived Neurotrophic Factor; EGF—Epidermal Growth Factor; MIF—Migration Inhibitory Factor; SCF—Stem Cell Factor; S100B—S100B protein, TNF-alpha—Tumor Necrosis Factor alpha, IL-8—Interleukin 8. tBDNF—total BDNF; mBDNF—mature BDNF; proBDNF—precursor BDNF; rBDNF—ratio (mBDNF/proBDNF).

**Table 2:** Significant correlations of studied proteins with the Temperament and Character Inventory dimensions and its subcomponents.

MDD + BD	R	*p*	MDD	R	*p*
IL-8 and self-directedness (SD)	0.36	0.003	IL-8 and reward-dependence (RD)	0.40	0.008
TNF-alpha and harm-avoidance (HA)	−0.32	0.01	TNF-alpha and reward-dependence (RD)	0.50	<0.00
TNF-alpha and reward-dependence (RD)	0.35	0.005	IL-8 and attachment (RD2)	0.44	0.0033
TNF-alpha and self-directedness (SD)	0.39	0.002	TNF-alpha and attachment (RD2)	0.53	<0.001
IL-8 and anticipatory worry (HA1)	−0.38	0.002	EGF and self-acceptance (SD4)	0.40	0.004
TNF-alpha and anticipatory worry (HA1)	−0.34	0.007	TNF-alpha and resourcefulness (SD3)	0.39	0.011
TNF-alpha and fatigability (HA4)	−0.33	0.008	s100b and empathy (C2)	−0.43	0.003
TNF-alpha and attachment (RD2)	0.45	<0.001	**BD**		
IL-8 and purposefulness (SD2)	0.37	0.002	EGF and novelty-seeking (NS)	0.59	0.002
IL-8 and enlightened second nature (SD5)	0.33	0.008	rBDNF and self-transcendence (ST)	0.56	0.006
TNF-alpha and purposefulness (SD2)	0.53	<0.001	tBDNF and persistence (PS)	0.58	0.003
TNF-alpha and resourcefulness (SD3)	0.38	0.002	mBDNF and persistence (PS)	0.7	<0.001
TNF-alpha and enlightened second nature (SD5)	0.38	0.002	rBDNF and disorderliness (NS4)	0.69	<0.001
s100b and empathy (C2)	−0.35	0.004	rBDNF and transpersonal identification (ST2)	0.6	0.003

Spearman’s rank correlation. Power ≥ 80%.

## Data Availability

The original contributions presented in the study are included in the article/Supplementary Material. Further inquiries can be directed to the corresponding author.

## Supplementary Materials

The following supporting information can be downloaded at: https://www.mdpi.com/article/10.3390/brainsci15020121/s1, Supplementary: Table S1 Correlations of protein levels with TCI Dimensions in MDD + BD, Table S2 Correlations of protein levels with TCI Dimensions in MDD, Table S3 Correlations of protein levels with TCI Dimensions in BD, Table S4 Correlations diagnosis conversion.
